# Implementation of a Comprehensive Curriculum in Personal Finance for Medical Fellows

**DOI:** 10.7759/cureus.2013

**Published:** 2018-01-01

**Authors:** Yuval D Bar-Or, Henry E Fessler, Dipan A Desai, Sammy Zakaria

**Affiliations:** 1 Carey Business School, Johns Hopkins University; 2 Department of Medicine, Johns Hopkins University School of Medicine; 3 Department of Medicine, University of Maryland Upper Chesapeake Medical Center

**Keywords:** graduate medical education, financial literacy, curriculum development research, financial exploitation

## Abstract

Introduction: Many residents and fellows complete graduate medical education having received minimal unbiased financial planning guidance. This places them at risk of making ill-informed financial decisions, which may lead to significant harm to them and their families. Therefore, we sought to provide fellows with comprehensive unbiased financial education and empower them to make timely, constructive financial decisions.

Methods: A self-selected cohort of cardiovascular disease, pulmonary and critical care, and infectious disease fellows (n = 18) at a single institution attended a live, eight-hour interactive course on personal finance. The course consisted of four two-hour sessions delivered over four weeks, facilitated by an unbiased business school faculty member with expertise in personal finance. Prior to the course, all participants completed a demographic survey. After course completion, participants were offered an exit survey evaluating the course, which also asked respondents for any tangible financial decisions made as a result of the course learning.

Results: Participants included 12 women and six men, with a mean age of 33 and varying amounts of debt and financial assets. Twelve respondents completed the exit survey, and all “Strongly Agreed” that courses on financial literacy are important for trainees. In addition, 11 reported that the course helped them make important financial decisions, providing 21 examples.

Conclusions: Fellows derive a significant benefit from objective financial literacy education. Graduate medical education programs should offer comprehensive financial literacy education to all graduating trainees, and that education should be provided by an unbiased expert who has no incentive to sell financial products and services.

## Introduction

Young physicians face substantial financial stress. According to the Association of American Medical Colleges, 76% of medical students in 2016 had educational debt, averaging $189,165, per physician at graduation [1]. Furthermore, many are considering or undertaking life decisions with financial implications, such as marriage, starting a family, or purchasing a home [2]. As a result, financial concerns affect career choice and contribute to stress during and after graduate medical education (GME) training [2-6].

Many physicians complete medical training lacking knowledge of basic finance [7-8]. This may lead them to suboptimal financial decisions, exposing physicians, their families, and their financial assets to excessive risk. Inadequate knowledge of personal finance also increases their vulnerability to brokers, advisors, and insurance agents who may not have a fiduciary responsibility to their physician clients.

For these reasons, medical students and GME trainees value unbiased financial literacy education [8-13]. However, attempts to provide this type of education have been limited, with rare examples of comprehensive courses covering personal finance and practice management [14]. One impediment to a more-widespread teaching of financial topics is the accessibility of knowledgeable and unbiased presenters [8]. While one program had a physician presenter who was also a certified financial planner [14], most lack similar internal resources. As a result, many GME programs rely on local, credentialed financial market professionals, whose teaching might conceal marketing goals. These raise concerns among students and trainees regarding the motivations of the presenters and the reliability of their advice [14].

Since financial literacy is essential for important life decisions and valued by GME trainees, we developed a course for residents and fellows covering all major areas of personal finance. Furthermore, all content was presented by a business school faculty member with specific expertise in teaching financial literacy to physicians and was not conflicted by having to sell financial products or services. Ultimately, we aimed to facilitate better, timelier financial decisions. This paper will describe the course development and its reception by trainees.

## Materials and methods

Program Development

The course developer and instructor (YDB) is a faculty member at the Johns Hopkins University Carey Business School with expertise and experience in teaching personal finance workshops customized for physicians. He initially developed a series of workshops, which included a range of prospective topics fundamental to financial literacy. The workshop curriculum was then revised in collaboration with physicians (HEF, DAD, SZ) who had roles in educating and supporting GME trainees. This pilot curriculum was then offered to fellows in cardiovascular disease at Johns Hopkins in January 2014 in 10 one-hour sessions over two weeks. Based on attendee feedback, future iterations of the curriculum only included eight topics (Table 1), omitting sessions on estate planning and the U.S. health care industry. These eight topics were then compressed into four two-hour blocks and scheduled in the autumn of 2016. Emails offering the revised curriculum were then sent to a new group of Johns Hopkins cardiovascular disease, infectious disease, and pulmonary and critical care fellows. Potential attendees were asked to confirm their interest by email. Lunch was provided, and the environment was purposely casual to encourage audience participation and questions.

Attendees were also provided with supplemental materials, including access to an online course containing over 50 videos, links to additional resources, summaries of retirement plans offered by the university, lists of questions to ask prospective financial advisors, and take-home spreadsheets and worksheets, to assist in developing household budgets and calculating net worth.

**Table 1:** Course topics

Course Topics
• Understanding the Time Value of Money; Interpreting Balance Sheets
• Basics of Investing, Risk, and Return
• Budgeting Strategies at Different Stages of Professional Development
• Managing Debt
• Understanding Psychological Barriers to Decision-Making
• Negotiating Employment Contracts
• Evaluating Needs and Selecting Insurance
• Dealing with Financial Advisors ​​​​​​​

Course Evaluation

A week before the 2016 course, a pre-course anonymous survey link was emailed to the ~25 cardiology, infectious disease, and pulmonary medicine fellows who had expressed interest in attending. The IRB-approved survey questions elicited demographic data and financial asset information. After course completion, another anonymous IRB-approved online survey was sent out by email to attendees. This included a link to questions addressing the importance of financial literacy to the responder and the value of the course, using a five-point Likert scale, ranging from strongly disagree (1) to strongly agree (5). Other questions asked whether tangible financial decisions were made because of the course and whether there was interest in other formats for this teaching. Respondents also had free-text options to describe course gaps or weaknesses.

All responses were collated and tabulated using survey software (Qualtrics, Provo, UT). Data were reported as numbers or percentages of respondents or means +/- SD, where appropriate. Responses to the Likert scale questions were converted to weighted averages. As this was a purely descriptive study without comparison groups, no statistical analyses were performed.

Of note, the course content and evaluation was reviewed by the Johns Hopkins Medicine Institutional Review Board and was deemed exempt from the requirement for informed consent when collecting survey and other evaluation data.

## Results

Respondent demographics and financial information

This course was offered to a total of 58 fellows in the three divisions, based on their potential availability to attend. A total of 21 attended at least one course session (36% of eligible attendees). There were 18 respondents (12 female) to the pre-course survey (Table 2). All were between the ages of 30-34, except for one who was 40. Out of these, 12 were married, all with employed spouses who generated an income for the household. Eight of the respondents had one child, with another having three children. Six respondents were single and six did not have children. An equal number of respondents (nine each) owned or rented homes. Most (16) owned cars, with one leasing and another neither owning nor leasing a vehicle.

**Table 2:** Demographic data among fellows completing the pre-survey. Data given as number (% of respondents) or mean +/- standard deviation.

Demographic Data	
Total Participants	18
Men	6 (33)
Women	12 (67)
Married	12 (67)
Respondents with Children	9 (50)
Dual Earner Household	12 (67)
Ages, Men	32 +/- 1.3 years
Ages, Women (12)	33 +/- 2.4 years
Owns Home	9 (50)
Owns Car	16 (89)
Leases Car	1
Does Not Have a Car	1

Ten participants provided estimates of household income, debt, and assets. Estimates varied widely, with participants reporting debts and assets ranging from <$40,000 to >$200,000. The range of household income was nearly as great.

Post-class survey responses

**Figure 1:**
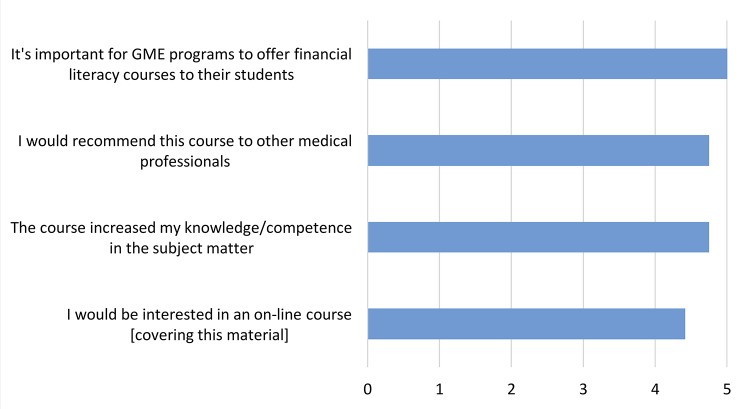
End-of-course survey responses. Attendees were asked to rate their agreement with each of the statements from strongly disagree (1) to strongly agree (5). The weighted mean of all responses is shown. N = 12

Twelve attendees completed the exit survey. Despite their other time commitments, respondents attended a mean of 70% of the class sessions. All “Strongly Agreed” with the statement: “It’s important for graduate medical education programs to offer such financial literacy courses to their students.” Respondents perceived that they learned the content and would recommend the course to other physicians (Figure 1). Many would be interested in receiving the material in other formats, including 11 (92%) interested in either online courses or webinars. Finally, 11 responded “Yes” to the question: “Has this course helped you to make any tangible financial decisions?” A follow-up question asking, “What was the nature of the decision?” elicited the responses summarized in Table 3. In total, 21 concrete financial decisions were made because of the course, with the greatest number related to retirement planning. Areas for improvement noted in free text included adding the opportunity for one-on-one consultation and more content on student loan repayment and forgiveness options.

**Table 3:** Financial decisions made as a result of attending the course, categorized into discrete financial domains.

Decision-Making Area	Number of Actions Taken
Retirement planning	7
Investing	5
Insurance coverage	3
Employment contracting	3
Debt management/Home mortgage	3
TOTAL	21

## Discussion

Despite demanding a significant time commitment from busy trainees, more than a third of invited fellows attended this financial literacy course. Our course was well-received by all attendees who completed the post-course survey and spurred most to make important financial decisions. While our survey sample size is small, we are confident that a course covering the major areas of personal finance and presented by an unbiased expert is important to almost all graduating GME trainees. In contrast to previous financial literacy interventions [7,14], we are the first to quantify the tangible actions resulting from broad financial literacy education among medical trainees. Most notably, we determined that many made major financial decisions, including setting up retirement plans, which may improve their long-term financial health.

The limited literature on the subject suggests that medical trainees often do not manage their finances judiciously. A 1999 survey of 612 residents in multiple fields at eight institutions found that residents had a mean debt/household income ratio of 2.48, compared to 1.06 for the age- and income-matched general public. Nearly 40% did not have a household budget, many were saving nothing for retirement, and 10% had credit card balances exceeding $10,000. Female residents were more financially conservative than males, and internal medicine residents were more conservative than general surgery residents [13]. A 2001 survey of 151 residents in urology found similar results, with 27% having less than $1000 in savings [12]. A survey of all emergency medicine residents conducted in 2001 (67% response rate) revealed that 82% received no financial planning education whatsoever during residency. Among those who received some instruction, in most cases, it was less than five hours [10]. We did not conduct a formal needs assessment among our learners, but a previously published assessment performed on surgical residents found widespread gaps [14]. Only 21% of the surgical residents reported having taken a course in personal finance, just one quarter felt they could manage their current finances, and only 7% felt prepared to manage their future finances. A study of radiation oncology residents revealed similar findings, with 75% feeling unprepared to handle future financial decisions [8].

Our curriculum was limited to personal finance topics shared by trainees in all fields, omitting topics related to office practice finance, coding, office personnel, and malpractice that have been covered in related curricula [14]. While important, these issues may have variable relevance depending on the trainee’s field and medical practice setting.

This study has several limitations. Although our findings are encouraging, the outcomes from our educational curriculum intervention remain preliminary and speculative. This was a small, single-institution study with a limited number of survey respondents. We do not have objective data on the financial knowledge of attendees, how it changed following the course, or compares to that of non-attendees. While most participants made financial decisions immediately after the course, it is unknown whether these will yield long-term financial benefits or would have been made eventually even without our course. Also, we have no information on the economic value of these financial decisions. Optimistically, informed attendees who received objective instruction in personal finance will make better decisions. Nevertheless, an annual follow-up would be valuable to determine if attendees continue to utilize the financial concepts they were taught.

We are also interested in optimizing the pedagogy for future iterations of this course. We may need to adjust time allocations for each topic or offer topics in alternative formats such as online. Multiple exposures to curriculum content, beginning in medical school and repeated in residency and fellowship [9], might improve the retention of financial literacy concepts and the enduring ability to make constructive decisions.

We had the advantage of a committed business school faculty member with particular expertise and interest in teaching this topic to medical trainees. That may not be the case at other institutions, where this gap has often been filled by insurance agents, financial advisors, or others. While they may offer excellent advice, it is generally unknown to the attendees whether they are also seeking clients or favoring products in which they have a financial interest. Our curriculum structure has fewer biases, as expressed by one of our attendees, who wrote: “I, like many other physicians, am extremely weary of financial ‘talks’ and so this unbiased presentation was exactly what was needed from a knowledgeable source.”

While it may be challenging to reproduce this curriculum at other institutions, collaboration or consultation with local business schools may identify faculty with similar skills. Courses can be scaled up to larger groups since the educational needs of residents, fellows, and junior faculty overlap. For paid instructors, this can allow costs to be shared across an institution. Although we found that in-person delivery facilitated learner engagement, learners also expressed interest in online formats. Shorter, less-comprehensive courses may also be effective. Dhaliwal and Chou found that a single, 90-minute investment seminar for internal medicine residents resulted in statistically significant changes in investment strategy among attendees compared to other residents [7]. Thus, while feasibility may be a challenge, we should not overlook this aspect of career development nor delegate it to those who may not always serve trainees’ best interests.

Medical students, residents, fellows, and junior faculty face enormous financial stresses. Over just a few years, they may accumulate large educational debts, enter long-term relationships, start families, move, purchase homes, and accept faculty appointments or private practice positions. Income fluctuates widely, likely more widely than during any other period in their lives. Extensive time and oversight are given to their clinical training and competence. Little is given to their financial training and competence. We believe that greater attention to their financial literacy will contribute to a greater sense of control over their future, with greater short- and long-term well-being.

## Conclusions

Graduating GME trainees perceive substantial benefits from objective financial literacy education. GME programs should offer comprehensive financial literacy education to all residents and fellows, provided by experts free of incentives to sell financial products and services. Further research should focus on graduating physicians’ long-term retention of financial knowledge and an assessment of their financial decision-making.
